# Long-Term Efficacy, Tolerability, and Renal Safety of Atazanavir/Ritonavir-based Antiretroviral Therapy in a Cohort of Treatment-Naïve Patients with HIV-1 Infection: the REMAIN Study

**DOI:** 10.1080/15284336.2015.1112494

**Published:** 2016-02-05

**Authors:** Eugénio Teófilo, Nuno Rocha-Pereira, Birger Kuhlmann, Antonio Antela, Heribert Knechten, Jesús Santos, Maria Jesús Jiménez-Expósito, on behalf of the REMAIN study group

**Affiliations:** ^a^ Department of Internal Medicine, Hospital Dos Capuchos, Lisboa, Portugal; ^b^ Centro Hospitalar de São João, Porto, Portugal; ^c^ Gemeinschaftspraxis Dres. Kuhlmann/Holm/Heiken, Hannover, Germany; ^d^ Infectious Diseases Unit, Hospital Clínico Universitario, Santiago de Compostela, Spain; ^e^ HIV Schwerpunktpraxis Dr. Heribert Knechten, Aachen, Germany; ^f^ Infectious Diseases Unit, Hospital Virgen de la Victoria, Malaga, Spain; ^g^ Bristol-Myers Squibb, Rueil-Malmaison, France

**Keywords:** HIV-1, ritonavir-boosted atazanavir, antiretroviral therapy, persistence, resistance, hyperbilirubinemia, observational study, estimated glomerular filtration rate

## Abstract

**Background:** Boosted protease inhibitors (PIs), including ritonavir-boosted atazanavir (ATV/r), are a recommended option for the initial treatment of HIV-1 infection based upon clinical trial data; however, long-term real-life clinical data are limited.

**Objective:** We evaluated the long-term use of ATV/r as a component of antiretroviral combination therapy in the real-life setting in the REMAIN study.

**Methods:** This was an observational cohort study conducted at sites across Germany, Portugal, and Spain. Retrospective historical and prospective longitudinal follow-up data were extracted every six months from medical records of HIV-infected treatment-naïve patients aged ≥ 18 years initiating a first-line ATV/r-containing regimen.

**Results:** Eligible patients (*n* = 517) were followed up for a median of 3.4 years. The proportion remaining on ATV/r at 5 years was 51.5% with an estimated Kaplan-Meier median time to treatment discontinuation of 4.9 years. Principal reasons for discontinuation were adverse events (15.9%; 8.9% due to hyperbilirubinemia) and virologic failure (6.8%). The Kaplan-Meier probability of not having virologic failure (HIV-1 RNA < 50 copies/mL) was 0.79 (95% CI: 0.75, 0.83) at five years. No treatment-emergent major PI resistance occurred. ATV/r was generally well tolerated during long-term treatment with no significant changes in estimated glomerular filtration rate over five years.

**Conclusions:** In a real-life clinical setting over five years, treatment-naïve patients with HIV-1 infection initiating an ATV/r-based regimen showed sustained virologic suppression, an overall treatment persistence rate of 51.5%, an absence of treatment-emergent major PI resistance mutations at virologic failure, a long-term safety profile consistent with that observed in clinical trials, and no significant decline in renal function.

## Introduction

Following the dramatic improvements in the survival of individuals with HIV-1 infection in the era of combined antiretroviral (ARV) therapy,^1^ HIV-1 infection is now considered a chronic disease requiring lifelong control with ARV therapy. Thus, achieving durable virologic suppression with ARV regimens that are well tolerated is now of major importance.^2^ However, data on long-term outcomes in patients receiving specific ARVs are limited.

Boosted protease inhibitors (PIs) possess a high genetic barrier to the development of viral resistance.^3^ In a meta-analysis of clinical trials, initial treatment with a regimen containing a boosted PI as the third agent was associated with less resistance within and across drug classes compared with initial treatment with a non-nucleos(t)ide reverse-transcriptase inhibitor as the third agent.^4^ These characteristics of boosted PIs have led to their inclusion as preferred agents in combination regimens for the initial treatment of HIV-1 infection in both US^5^ and EU^6^ treatment guidelines. Randomized controlled trials of up to four years’ duration in treatment-naïve patients with HIV-1 infection have shown that regimens containing ritonavir-boosted atazanavir (ATV/r) are an effective and well-tolerated treatment.^7−9^ Although complementary real-life observational data evaluating ATV/r-based regimens are available,^10−12^ these data are limited, and thus additional data with a longer follow-up duration and in different clinical settings are required.

The aim of the REMAIN observational cohort study was to evaluate long-term outcomes in ARV-naïve patients initiating ATV/r-containing regimens in a real-life clinical setting over a maximum follow-up period of five years.

## Methods

### Study design

The REMAIN study was a non-comparative, observational study conducted at a total of 51 sites across Germany, Portugal, and Spain (ClinicalTrials.gov identifier: NCT01236235). This study was conducted in accordance with the International Society for Pharmacoepidemiology Guidelines for Good Epidemiology Practices, the ethical principles originating from the Declaration of Helsinki, and local regulatory requirements. Local ethical committees approved the protocol, amendments, and the patient informed consent form. Written informed consent from all participants was obtained.

### Patients

Consecutive patients attending the investigating center(s) with HIV-1 infection aged ≥ 18 years who were ARV treatment-naïve when an ATV/r-based regimen was initiated were selected for this study. The ATV/r-based regimen, which included at least two nucleos(t)ide reverse transcriptase inhibitors, must have been initiated between 1 February 2008 and 31 July 2010, and patients must have been seen in routine outpatient consultations between 1 January 2011 and 31 March 2012. Retrospective historical and prospective longitudinal follow-up data were extracted every six months from the medical records with follow-up continuing until 31 July 2013 (the protocol was amended in July 2012 to allow for a one-year extension to the follow-up period). Patients with more than four weeks of prior ARV exposure or who were participating in an atazanavir (ATV) clinical trial were excluded.

### Study endpoints

#### Persistence on treatment

The primary endpoint was the proportion of patients remaining on an ATV/r-based regimen over time. Other endpoints examining treatment persistence included: (1) the probability of remaining on treatment and time to treatment discontinuation assessed using the Kaplan-Meier method; (2) baseline factors associated with treatment discontinuation, assessed using univariate and multivariate Cox proportional hazards models; and (3) the overall proportion of patients discontinuing treatment and reasons given by the investigator for treatment discontinuation.

#### Virologic and immunologic outcomes

Virologic and immunologic outcomes included: (1) time to virologic failure, defined as two consecutive HIV-1 RNA values of ≥ 50 copies/mL, or as one HIV-1 RNA value of ≥ 50 copies/mL followed by discontinuation, regardless of the reason, more than six months from ATV/r initiation (virologic failure was also analyzed using a ≥ 500 copies/mL cut-off); (2) the proportion of patients with virologic failure (as defined above) and its inverse, virologic response; (3) treatment-emergent new major or minor PI resistance mutations, defined using the International Antiviral Society–USA classification system,^13^ assessed at the time of virologic failure; and (4) HIV-1 RNA changes and CD4 cell count changes over time.

#### Long-term safety and tolerability

The long-term safety and tolerability profile of ATV/r was assessed by the following parameters: (1) the frequency of adverse events (AEs) and AEs leading to discontinuation; (2) laboratory abnormalities graded according to the Division of AIDS system;^14^ and (3) renal safety as measured by the frequency of renal-specific AEs (such as nephrolithiasis or renal failure) and by changes in glomerular function over time (assessed by calculating estimated glomerular filtration rate [eGFR] using the Modification of Diet in Renal Disease equation^15^).

### Statistical analysis

For the analysis of time-to-event data, the Kaplan-Meier method was considered to be the most appropriate technique. Patients with missing data or who were lost to follow-up were censored. Patients still participating in the study as of December 2012, but who did not provide their consent to continue participation in the one-year extension to the follow-up period during 2013, were considered as having discontinued ATV/r.

Baseline factors potentially associated with time to treatment discontinuation were investigated using Cox proportional hazards models. Gender, viral load at baseline (HIV-1 RNA < or ≥ 100,000 copies/mL), and co-infection with hepatitis B or C virus were entered into all models. Other factors, including country, age at initiation, disease duration, CD4 cell count, mode of HIV acquisition, and history of opportunistic infections, were preselected on the basis of a significance level of 0.2 in univariate Cox models provided excessive collinearity was not present. Pairwise interactions were tested in the final model and further selected for retention or exclusion by backward elimination.

For the statistical analysis of changes in HIV-1 RNA, CD4 cell counts, and eGFR over time, mixed-models repeated measures (MMRM) analysis was considered to be a more appropriate method for imputing missing data than the last observation carried forward (LOCF) method because MMRM generates estimates that are valid for most types of missing data, whereas LOCF does not.^16^

The long-term safety profile of ATV/r treatment was evaluated by collecting data on reported AEs (as listed by study investigators without predefinition) and laboratory abnormalities. AE data were collected and analyzed using descriptive statistics (counts, proportions, and percentages) for all patients up to the point of ATV/r treatment discontinuation or, for those patients who remained on treatment at the end of the study, until the end of the follow-up period.

## Results

### Patients

**Figure 1:**
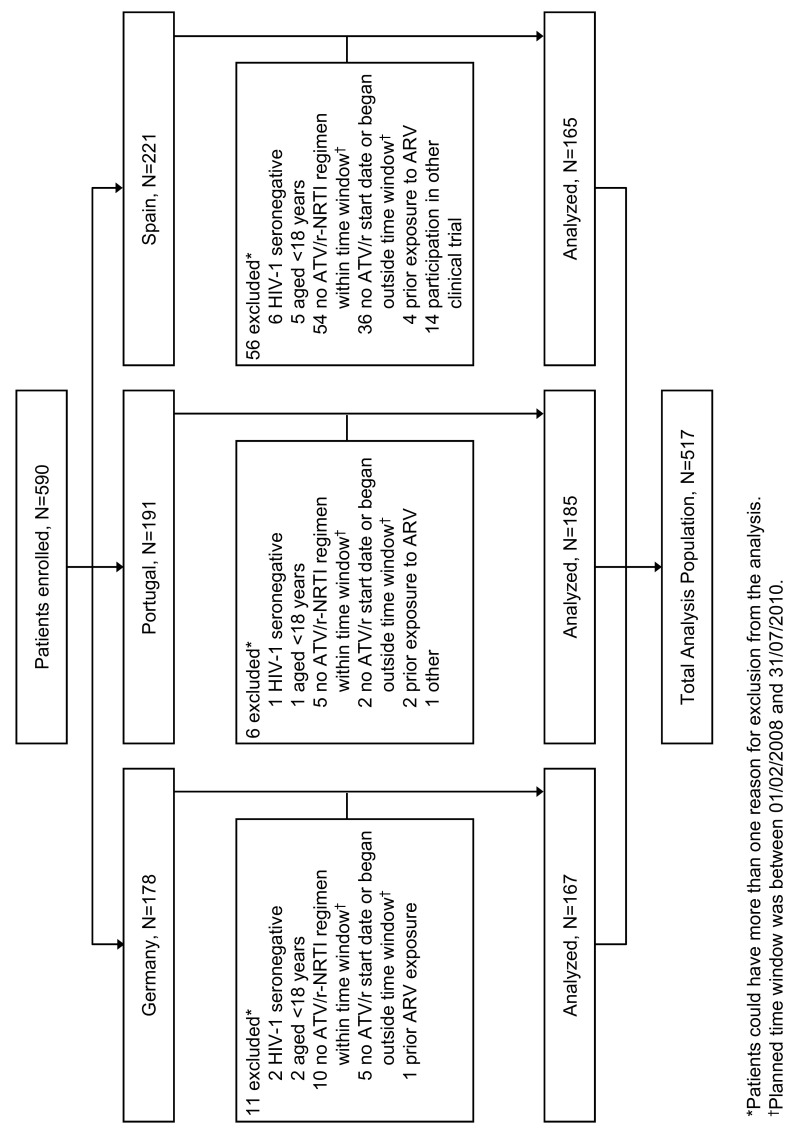
Patient disposition from enrollment to eligibility. ARV, antiretroviral; ATV/r, ritonavir-boosted atazanavir; NRTI, nucleos(t)ide reverse transcriptase inhibitors.

Of 590 patients enrolled, 517 patients were eligible for inclusion in the study analyses. Details of patient disposition from enrollment to study analysis eligibility are summarized in Figure1. Demographic and baseline characteristics are presented in Table 1. At baseline, median age was 40 years, the majority were men (76.0%), most patients were white (89.4%), and the route of transmission was predominantly sexual, with 16.9% acquiring HIV-1 infection from intravenous drug use. Centers for Disease Control and Prevention Class C AIDS was present in 18.8% of patients, HIV-1 RNA ≥ 100,000 copies/mL in 43.2%, CD4 count < 200 cells/mm^3^ in 38.2% and < 50 cells/mm^3^ in 10.4%, and hepatitis C virus co-infection in 18.6%. The majority of patients were receiving a fixed-dose combination of either tenofovir disoproxil fumarate/emtricitabine (TDF/FTC; 81.8%) or abacavir/lamivudine (ABC/3TC; 9.3%) as backbone ARV therapy. Patients were followed-up for a median period of 2.0 years (interquartile range [IQR]: 1.3 to 2.7) during the retrospective period and a median period of 1.5 years (IQR: 1.3 to 2.1) during the prospective period, giving a total median duration of exposure to ATV/r of 3.4 years (IQR: 1.8 to 4.3).

**Table 1:** Demographic and baseline characteristics

	Germany (*N* = 167)	Portugal (*N* = 185)	Spain (*N* = 165)	Overall (*N* = 517)
Age at ATV/r initiation, median (IQR), years	41 (32, 48)	41 (35, 51)	39 (33, 45)	40 (33, 48)
Male gender, n (%)	136 (81.4)	128 (69.2)	129 (78.2)	393 (76.0)
Race, n (%)				
White	149 (89.2)	166 (89.7)	147 (89.1)	462 (89.4)
Black or African-American	11 (6.6)	19 (10.3)	8 (4.8)	38 (7.4)
Other	7 (4.2)	0	10 (6.1)	17 (3.3)
Route of transmission, n/N# (%)				
Heterosexual	13/126 (10.3)	80/179 (44.7)	48/146 (32.9)	141/451 (31.3)
Men who have sex with men	91/126 (72.2)	27/179 (15.1)	62/146 (42.5)	180/451 (39.9)
Sexual not categorized	12/126 (9.5)	33/179 (18.4)	7/146 (4.8)	52/451 (11.5)
Intravenous drug use	9/126 (7.1)	39/179 (21.8)	28/146 (19.2)	76/451 (16.9)
Other	1/126 (0.8)	0	1/146 (0.7)	2/451 (0.4)
CDC Class C AIDS, n/N# (%)	21/162 (13.0)	47/185 (25.4)	26/154 (16.9)	94/501 (18.8)
Hepatitis C co-infection, n/N# (%)	19/152 (12.5)	42/185 (22.7)	31/158 (19.6)	92/495 (18.6)
HIV-1 RNA ≥100,000 copies/mL, n/N# (%)	67/155 (43.2)	81/181 (44.8)	65/157 (41.4)	213/493 (43.2)
CD4 count, n/N# (%)				
<200 cells/mm^3^	56/156 (35.9)	84/179 (46.9)	47/155 (30.3)	187/490 (38.2)
<50 cells/mm^3^	10/156 (6.4)	27/179 (15.1)	14/155 (9.0)	51/490 (10.4)
Time of ATV/r exposure, median (IQR), months	44.0 (27.4, 54.1)	40.8 (20.6, 51.4)	39.5 (15.8, 49.1)	41.0 (21.0, 51.4)
Backbone antiretroviral therapy, n (%)				
* Fixed*-*dose combinations*				
TDF/FTC (Truvada^®^)	152 (91.0)	147 (79.5)	124 (75.2)	423 (81.8)
ABC/3TC	11 (6.6)	23 (12.4)	14 (8.5)	48 (9.3)
ZDV/3TC	2 (1.2)	0 (0)	3 (1.8)	5 (1.0)
* Other antiretrovirals*				
Abacavir	0 (0)	2 (1.1)	4 (2.4)	6 (1.2)
Emtricitabine	1 (0.6)	13 (7.0)	18 (10.9)	32 (6.2)
Lamivudine	1 (0.6)	1 (0.5)	5 (3.0)	7 (1.4)
Nevirapine	1 (0.6)	1 (0.5)	0 (0)	2 (0.4)
Ritonavir	20 (12.0)	1 (0.5)	0 (0)	21 (4.1)
Tenofovir	1 (0.6)	13 (7.0)	20 (12.1)	34 (6.6)
Zidovudine	1 (0.6)	1 (0.5)	1 (0.6)	3 (0.6)
Baseline eGFR,^a^ median (IQR), mL/min	102.1 (87.5, 116.4)	105.1 (92.7, 125.4)	99.8 (85.1, 119.8)	102.1 (88.9, 120.6)

*Note: N* = total number of patients; *n* = number of patients with characteristic; *N*# = total number of patients with available data for each characteristic.

^a^ Available in 455 patients – eGFR calculated using Modification of Diet in Renal Disease formula. ABC/3TC, abacavir/lamivudine; ATV/r, ritonavir-boosted atazanavir; CDC, Centers for Disease Control and Prevention; eGFR, estimated glomerular filtration rate; IQR, interquartile range; TDF/FTC, tenofovir disoproxil fumarate/emtricitabine; ZDV/3TC, zidovudine/lamivudine.

### Persistence on treatment

**Figure 2:**
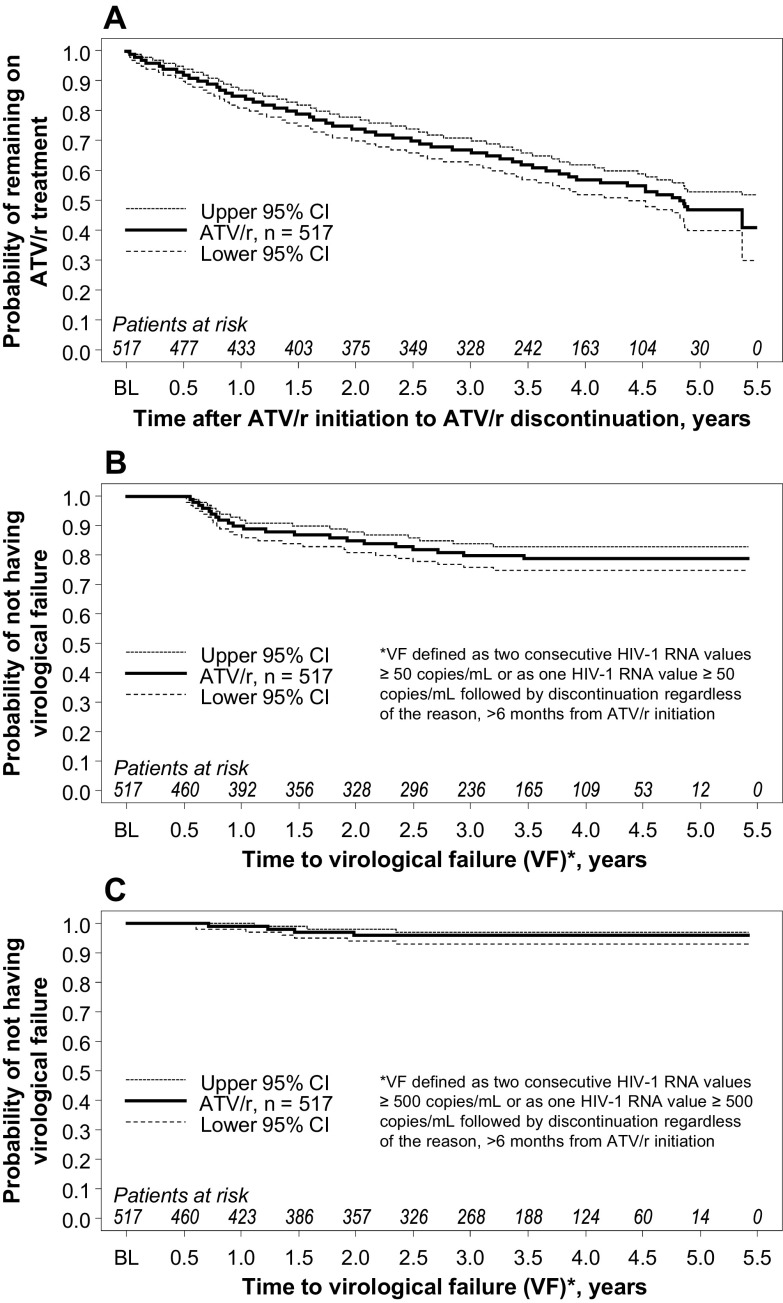
Time to discontinuation of treatment (A) and time to virologic failure using ≥ 50 copies/mL cut-off (B) and using ≥ 500 copies/mL cut-off (C). ATV/r, ritonavir-boosted atazanavir; BL, baseline; CI, confidence interval.

At the end of the follow-up period, after a mean follow-up duration of 3.1 (standard deviation: 1.5) years, a total of 264 patients (51.5%; 95% confidence interval [CI]: 47.0, 55.9) remained on an ATV/r-based regimen. Accounting for censored observations by survival analysis techniques (i.e. patients lost to follow-up and missing data), the probabilities of remaining on an ATV/r-based regimen were 0.85 (95% CI: 0.81, 0.87), 0.74 (95% CI: 0.70, 0.78), 0.67 (95% CI: 0.62, 0.71), 0.57 (95% CI: 0.52, 0.62), and 0.47 (95% CI: 0.40, 0.53) for the first, second, third, fourth, and fifth years, respectively (Figure 2A). The Kaplan-Meier estimated median time to treatment discontinuation was 4.9 years (95% CI: 4.5, upper limit non-estimable).

On univariate Cox proportional hazards analysis, country of origin and a history of opportunistic infection at baseline were associated with an increased risk of treatment discontinuation (Table 2). On multivariate analysis, only country of origin remained significantly associated; specifically, patients from Germany had a reduced risk of treatment discontinuation versus those from Spain (Table 2). None of the factors forced in the model (gender, baseline HIV-1 RNA level, or hepatitis B or C virus co-infection status) showed a statistically significant effect in the final model (*p* > 0.10).

**Table 2:** Univariate and multivariate Cox proportional hazards models for time to discontinuation of an ATV/r-based regimen

Univariate analysis				
*Baseline covariate*	*Continuous or covariate subcategories*	*HR* (95*%* CI)^a^	*P-value* (*HR =* 1)^b^	*P-value Type 3 Test* (*β =* 0)^c^
Gender	Males relative to females	0.83 (0.62, 1.12)	0.22847	0.22847
HIV-1 RNA level	≥100,000 relative to <100,000 copies/mL	1.21 (0.92, 1.58)	0.17039	0.17039
Hepatitis B virus co-infection	Present relative to not present	0.73 (0.36, 1.49)	0.38843	0.44477
	Unknown relative to not present	0.77 (0.47, 1.29)	0.32629	
Hepatitis C virus co-infection	Present relative to not present	0.99 (0.71, 1.40)	0.96565	0.77344
	Unknown relative to not present	0.77 (0.38, 1.57)	0.47372	
Country	Germany relative to Spain	0.65 (0.46, 0.90)	0.00913	0.03113
	Portugal relative to Spain	0.79 (0.58, 1.07)	0.12777	
Age at ATV/r initiation (years)	<50 relative to ≥50	0.86 (0.63, 1.16)	0.32175	0.32175
	Continuous	1.01 (0.99, 1.02)	0.30526	0.30526
Disease duration^d^ (years)	Continuous	1.01 (0.97, 1.04)	0.73004	0.73004
Mode of HIV acquisition	IVDU relative to other	1.13 (0.78, 1.64)	0.52113	0.53152
	Unknown relative to other	1.22 (0.83, 1.78)	0.3074	
CD4 count at baseline	<200 copies/mm^3^ relative to ≥350 copies/mm^3^	1.18 (0.83, 1.69)	0.35261	0.26431
	200–350 copies/mm^3^ relative to ≥350 copies/mm^3^	0.92 (0.64, 1.32)	0.64589	
	Continuous	1.00 (1.00, 1.00)	0.19211	0.19211
Opportunistic infection history	Present relative to not present	1.38 (1.01, 1.89)	0.04275	0.01503
	Unknown relative to not present	2.29 (1.12, 4.65)	0.02273	
				
Multivariate analysis^e^				
*Baseline covariate*	*Continuous or covariate subcategories*	*Adjusted HR (95% CI)*^f^	*P-value (adjusted HR* = 1)^g^	*P-value Type 3 Test (β =* 0)^c^
Gender	Males relative to females	0.77 (0.56, 1.06)	0.112	0.112
HIV-1 RNA level	≥100,000 relative to <100,000 copies/mL	1.26 (0.96, 1.66)	0.101	0.101
Hepatitis B virus co-infection	Present relative to not present	0.90 (0.44, 1.84)	0.771	0.949
	Unknown relative to not present	0.95 (0.47, 1.90)	0.881	
Hepatitis C virus co-infection	Present relative to not present	1.05 (0.74, 1.49)	0.796	0.611
	Unknown relative to not present	0.61 (0.22, 1.71)	0.343	
Country	Germany relative to Spain	0.67 (0.47, 0.95)	0.024	0.049
	Portugal relative to Spain	0.74 (0.54, 1.03)	0.071	

^a^ Crude (unadjusted) HR of time to discontinuation of ATV/r-based regimen (in months).

^b^ *P*-value tests HR = 1;

^c^ Tests the overall effect of the factor (global null hypothesis: *β* = 0). Factors with a type 3 test p-value < 0.2 were eligible for entering for inclusion in the starting multivariate model provided excessive collinearity was not present.

^d^ Since ATV/r initiation.

^e^ Gender, category of viral load at baseline, and co-infection with hepatitis B or C virus at baseline were forced into all models. Other cofactors/covariates were selected if they were significant at level 0.2 in the univariate Cox model (type 3 test), displayed limited collinearity, and were retained following a forward selection process. The number of patients able to be included in the final model was 493/517 (95.4%).

^f^ Covariate adjusted HR of time to discontinuation of ATV/r-based regimen (in months).

^g^ *P-*value tests adjusted HR = 1.

ATV/r, ritonavir-boosted atazanavir; CI, confidence interval; HR, hazard ratio.

Overall, 223/517 patients (43.1%) had discontinued ATV/r by the end of the follow-up period for up to five years, with a lower rate in Germany than in Portugal or Spain (Table 3). The most frequent reasons given by the investigator included AEs in 15.9% of patients, medical decision or regimen simplification in 9.5%, and virologic failure in 6.8% (Table 3). Overall, 26/517 patients (5.0%) were lost to follow-up for up to five years and 27/517 patients (5.2%) did not provide consent to continue in the one-year extension period and were therefore considered as having discontinued ATV/r as per the protocol amendment.

**Table 3:** Reasons given by the investigator for discontinuation

	Germany (*N* = 167)	Portugal (*N* = 185)	Spain (*N* = 165)	Overall (*N* = 517)
ATV/r discontinuation, n (%)	62 (37.1)	78 (42.2)	83 (50.3)	223 (43.1)
Reasons for discontinuation,^a^ n (%)				
Virologic failure^b^	5 (3.0)	19 (10.3)	11 (6.7)	35 (6.8)
Adverse event	34 (20.4)	17 (9.2)	31 (18.8)	82 (15.9)
Nausea	2 (1.2)	1 (0.5)	2 (1.2)	5 (1.0)
Vomiting	1 (0.6)	3 (1.6)	3 (1.8)	7 (1.4)
Renal failure	3 (1.8)	0	2 (1.2)	5 (1.0)
Hyperbilirubinemia-related^c^	19 (11.4)	10 (5.4)	17 (10.3)	46 (8.9)
Poor/non-compliance	4 (2.4)	9 (4.9)	2 (1.2)	15 (2.9)
Concomitant disease	1 (0.6)	0	0	1 (0.2)
Patient request	8 (4.8)	2 (1.1)	3 (1.8)	13 (2.5)
Drug interaction	2 (1.2)	3 (1.6)	3 (1.8)	8 (1.5)
Medical decision/regimen simplification	7 (4.2)	21 (11.4)	21 (12.7)	49 (9.5)
Consent unavailable for 1-year extension	2 (1.2)	11 (5.9)	14 (8.5)	27 (5.2)
Other	1 (0.6)	3 (1.6)	0	4 (0.8)
Unknown	0	1 (0.5)	1 (0.6)	2 (0.4)

^a^ Patients could discontinue ATV treatment for more than one reason.

^b^ Virologic failure reported by the investigator as reason for discontinuation.

^c^ The number of patients with at least one hyperbilirubinemia-related discontinuation was calculated: four patients (three from Germany and one from Portugal) each had two hyperbilirubinemia-related reasons recorded; therefore, four of these reasons were excluded from this calculation.

### Virologic and immunologic outcomes

Using the <50 copies/mL cut-off, the Kaplan-Meier estimated probabilities of not having virologic failure were 0.90 (95% CI: 0.86, 0.92), 0.85 (95% CI: 0.81, 0.88), 0.80 (95% CI: 0.76, 0.84), 0.79 (95% CI: 0.75, 0.83), and 0.79 (95% CI: 0.75, 0.83) for the first, second, third, fourth, and fifth years, respectively (Figure 2B). Using the <500 copies/mL cut-off, the Kaplan-Meier estimated probabilities of not having virologic failure were 0.99 (95% CI: 0.98, 1.00) for the first year, 0.96 (95% CI: 0.94, 0.98) for the second year, and 0.96 (95% CI: 0.93, 0.97) for the third, fourth, and fifth years (Figure 2C).

Eighty-five patients met the definition of virologic failure based upon the ≥50 copies/mL cut-off during the period of the study, representing a virologic response rate of 83.6%. At the time of virologic failure, 19 of these 85 patients (22.4%) had treatment-emergent minor PI resistance mutations, but no patient had treatment-emergent major PI resistance mutations.

**Figure 3:**
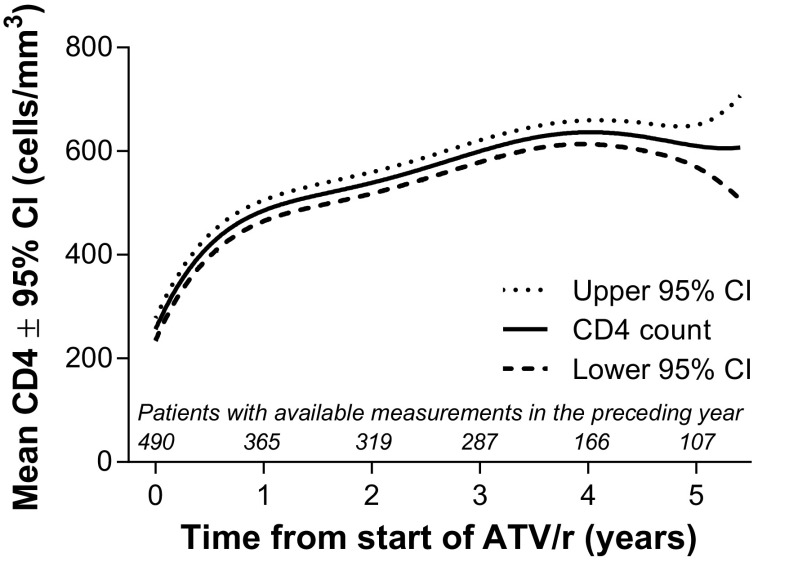
Mixed-model repeated measures estimates of mean CD4 count over time. ATV/r, ritonavir-boosted atazanavir; CI, confidence interval.

Mean HIV-1 RNA levels significantly decreased by 269,534 copies/mL (95% CI: 266,109, 272,960) over the first six months after ATV/r initiation (the associated proportion with HIV-1 RNA < 50 copies/mL at six months was 82.7%), with little change thereafter (MMRM with adjustment for baseline value, *p* < 0.0001). In parallel, immunologic recovery was observed; the median CD4 cell count at the last available assessment was 549 cells/mm^3^ (IQR: 376 to 730), representing a median increase from baseline of 331 cells/mm^3^ (IQR: 158 to 466). Using a statistical method robust to data missing at random, MMRM analysis of CD4 cell counts showed progressive and significant increases over time (*p* < 0.0001), reaching 610 cells/mm^3^ at five years (95% CI: 569, 651) (Figure 3).

### Long-term safety and tolerability

ATV/r was generally well tolerated during long-term treatment. Overall, at least one AE was reported in 48.5% of patients. Serious AEs were reported in two patients (cholecystitis and vaginal bleeding), which did not result in any change to the ATV/r-based regimen.

Treatment-emergent clinical AEs (of any grade) and laboratory AEs (of grade 3–4 severity), regardless of causality, are shown in Table 4. As expected from the mechanism of action of ATV, AEs of hyperbilirubinemia were reported in 29.4% and Grade 3–4 bilirubin elevations in 37.8% of patients. Gastrointestinal AEs were uncommon, occurring in ≤2.5% of patients. Grade 3–4 elevations in lipid parameters occurred in ≤6.5% of patients.

**Table 4:** Selected treatment-emergent clinical and laboratory AEs (regardless of causality): incidence in the overall population of eligible patients (N = 517)

Total and selected clinical AEs (any grade)	*n* (%)
Patients with any AE	251 (48.5)
Diarrhea	10 (1.9)
Nausea	10 (1.9)
Vomiting	13 (2.5)
Hyperbilirubinemia	152 (29.4)
Jaundice	22 (4.3)
Ocular icterus	10 (1.9)
Renal and urinary disorders	17 (3.3)
Chromaturia	1 (0.2)
Dysuria	1 (0.2)
Nephrolithiasis	8 (1.5)
Pollakiuria	1 (0.2)
Renal colic	2 (0.4)
Renal failure	5 (1.0)
Laboratory AEs (Grade 3–4)	*n*^a^ / N^b^ (%)
ALT (> 5.1 × ULN)	18/504 (3.6)
AST (> 5.1 × ULN)	5/504 (1.0)
Total cholesterol (≥ 300 mg/dL)	14/496 (2.8)
Triglycerides (≥ 751 mg/dL)	8/497 (1.6)
LDL-cholesterol (≥ 190 mg/dL)	28/434 (6.5)
Total bilirubin (> 2.5 × ULN)	188/497 (37.8)
Creatinine (> 2 × ULN)	3/499 (0.6)

n^a^ = number of patients with at least one laboratory value above thresholds whilst on treatment. N^b^ = patients with available laboratory parameter values while on treatment. For laboratory abnormalities, the highest post-baseline value was considered. Clinical AEs reported by investigator were coded and grouped using the latest version of the Medical Dictionary for Regulatory Activities (MedDRA). Patients with more than one AE with the same preferred term were counted once for that term. Toxicity grades were defined according to the Division of AIDS Table for Grading the Severity of Adult and Pediatric Adverse Events. AE, adverse event; ALT, alanine aminotransferase; AST, aspartate aminotransferase; LDL, low-density lipoprotein; ULN, upper limit of normal.

Within the group of patients discontinuing because of AEs, few patients (<1.5%) discontinued for gastrointestinal or renal reasons with the most common reason being hyperbilirubinemia-related (Table 3). Hyperbilirubinemia-related discontinuations are presented in detail in Table 5. Four patients were reported as each having two hyperbilirubinemia-related reasons for discontinuation. After excluding the four duplicated reasons, 46/517 patients (8.9%) were reported by the investigator as having discontinued for at least one hyperbilirubinemia-related reason during five years of follow-up. Among these hyperbilirubinemia-related discontinuations, investigators gave a raised blood bilirubin (defined as reporting one of three terms: ‘hyperbilirubinemia’, ‘blood bilirubin abnormal’, or ‘blood bilirubin increased’) as the reason in 21/46 patients (45.7%) and a clinical reason (defined as reporting ‘jaundice’, ‘ocular icterus’, or ‘yellow skin’) in 25/46 patients (54.3%). Not all patients had a confirmatory bilirubin measurement prior to discontinuation; for those that had, 13/517 patients (2.5%) were discontinued despite having Grade 1–2 elevations. By five years, the rate of discontinuation due to hyperbilirubinemia with a confirmed Grade 1–4 bilirubin measurement was 34/46 (6.6%) and with a confirmed Grade 3–4 bilirubin elevation was 21/46 (4.1%). Hyperbilirubinemia-related discontinuations varied by country, occurring most commonly in Germany, where ocular icterus was reported as the reason for discontinuation in seven out of the eight patients with this AE.

**Table 5:** Patients discontinued due to hyperbilirubinemia: reasons given by the investigator versus confirmed grading from bilirubin measurement

Data: n (% of 46) [% of 517]	Reason given for discontinuation by the investigator				
Confirmed Grade	Raised blood bilirubin^a^	Jaundice	Ocular icterus	Yellow skin	Total
Grade 1–2 hyperbilirubinemia	6 (13) [1.2]	3 (6.5) [0.6]	4 (8.7) [0.8]	(0) [0]	13 (28.3) [2.5]
Grade 3–4 hyperbilirubinemia	12 (26.1) [2.3]	6 (13) [1.2]	3 (6.5) [0.6]	(0) [0]	21 (45.7) [4.1]
Measurement unavailable	3 (6.5) [0.6]	7 (15.2) [1.4]	1 (2.2) [0.2]	1 (2.2) [0.2]	12 (26.1) [2.3]
Total	21 (45.7) [4.1]	16 (34.8) [3.1]	8 (17.4) [1.5]	1 (2.2) [0.2]	46 (100) [8.9]

^a^ Terms recorded by the investigator for this category included, ‘hyperbilirubinemia’, ‘blood bilirubin abnormal’, and ‘blood bilirubin increased’.

**Figure 4:**
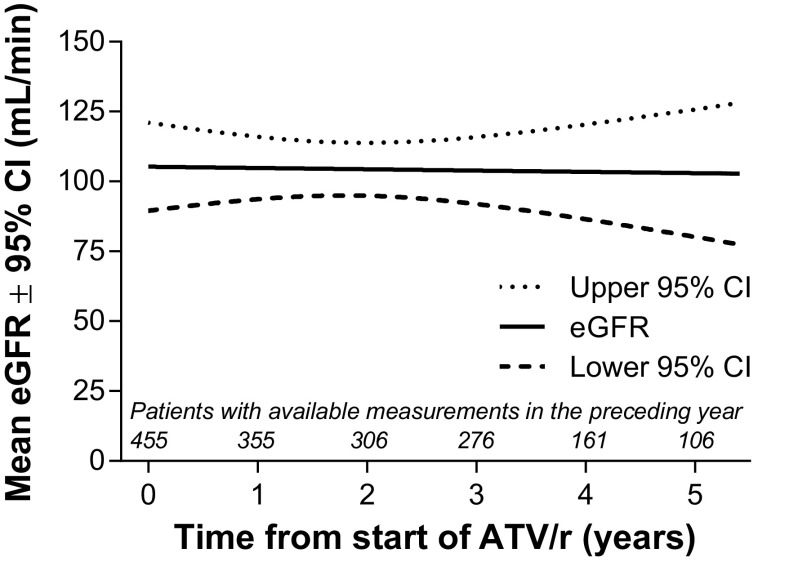
Mixed-model repeated measures estimates of mean estimated glomerular filtration rate (eGFR) over time. ATV/r, ritonavir-boosted atazanavir; CI, confidence interval.

Overall, renal/urinary disorders occurred in 17 patients (3.3%). Grade 3–4 creatinine elevations were rare (<1%). The incidence of nephrolithiasis in the overall population was 0.6% per patient-year of exposure. Renal failure, which also led to treatment discontinuation in all cases, occurred in five patients (<1% of the overall population), one of whom was also receiving concomitant TDF/FTC. At the last available patient assessment, median eGFR for the overall patient population was 95.3 mL/min (IQR: 81.4 to 113.8), representing a median change from baseline of −4.7 mL/min (IQR: –21.3 to 0.0). Using a statistical method robust to data missing at random, MMRM analysis of eGFR showed no significant change over time with treatment (Figure 4); mean change from baseline at five years in eGFR was −1.3 mL/min (95% CI: −30.3, 27.8). eGFR > 90 mL/min at baseline declined to < 60 and < 30 mL/min in 11 (2.6%) and one patient (<1%), respectively.

## Discussion

In this large European cohort of treatment-naïve patients with HIV-1 infection commencing ATV/r-based regimens, the probability of remaining virologically suppressed (HIV-1 RNA < 50 copies/mL) for up to 5 years was high at 80%, confirming previous findings from ATV/r clinical trials.^7−9^ No major PI resistance mutations were observed at virologic failure and no unexpected AEs were reported. Overall, the incidence of renal disorders was low and similar to that observed in clinical trials,^7−9,17^ and no significant changes in eGFR were noted over five years of treatment with an ATV/r-based regimen.

Persistence with ATV/r-based regimens over time was good, with 51.5% of patients remaining on treatment up to five years in the current study; this compares favorably with the results of other cohort studies examining discontinuation rates with several different first-line ARV-regimens^10−12^ and in other cohort studies in treatment-experienced patients.^18,19^ In the Swiss HIV Cohort Study, comparing seven initial ARV treatment regimens, rates of treatment modification one year after the start of treatment were lowest for ATV/r plus TDF/FTC (24.1%) and highest for ritonavir-boosted lopinavir plus zidovudine/lamivudine (70.7%).^10^ Similarly, in a comparative five-year cohort study of first-line combination ARV therapy, ATV/r showed greater persistence (1016 days) than efavirenz (974 days) or ritonavir-boosted lopinavir (382 days).^11^ In a pooled analysis of 18 cohort studies conducted in Europe and North America (*n* = 21,801), treatment modification and interruption rates were high (51% after 2.3 years); however, patients taking ATV had lower rates of changing drug class compared with patients on efavirenz, whereas patients on lopinavir or nevirapine had higher rates of interruption.^12^ In a retrospective European cohort study, in treatment-experienced patients (*n* = 1,294) who switched to an ATV/r-containing regimen, a treatment discontinuation rate of 21.2% over the first year was reported, with a probability of remaining on treatment after three years of 0.56 (95% CI: 0.52, 0.60) for patients with baseline virologic suppression (<500 copies/mL).^18^ In a subsequent analysis of data from this study by gender, treatment discontinuation rates at one year were 40.0 and 52.1% for the male and female patients, respectively; after three years, the probability of male patients remaining on ATV/r treatment (0.58; 95% CI: 0.54, 0.61) was higher than for female patients (0.46; 95% CI: 0.40, 0.52), based on Kaplan-Meier analysis.^19^

AEs were reported as reasons for treatment discontinuation in approximately 16% of patients in the current study, with the majority of individual events occurring in <5% of patients. Consistent with ATV inhibition of uridine 5’-diphospho-glucuronosyltransferase (UGT),^20^ the enzyme responsible for bilirubin glucuronidation, hyperbilirubinemia was reported as a clinical or Grade 3–4 laboratory event (total bilirubin elevation > 2.5 x upper limit of normal) in 29.4% and 37.8% of patients, respectively. Discontinuation due to hyperbilirubinemia by five years occurred in 8.9% of patients overall, in 6.6% with a confirmed bilirubin measurement, and in 4.1% with Grade 3–4 bilirubin elevations. Although the rate of Grade 3–4 hyperbilirubinemia AEs in the current study (37.8%) compared favorably with the rate in a similar observational cohort study in treatment-experienced patients switched to an ATV/r-based regimen (61.0%),^18^ the rate of discontinuation due to hyperbilirubinemia was rare in this latter study (0.9%).^18^ However, the latter study was entirely retrospective in design, whereas the current study included a prospective component, which may have led to underreporting of the reasons for treatment discontinuation in the latter study. Despite rates of Grade 3–4 hyperbilirubinemia in the current study (37.8%) being lower than those in major randomized controlled trials (44–58%),^7−9,21−25^ discontinuation due to hyperbilirubinemia was higher in the current study (8.9%) compared with these randomized controlled trials (0–7.8%).^7−9,21−25^ When considering discontinuation due to clinical jaundice (includes ‘jaundice’, ‘ocular icterus’, and ‘yellow skin’ as reasons), the rate in the current study (4.8%) was similar to that reported in the AIDS Clinical Trials Group A5257 study (5.0%).^23^ Rates of discontinuation due to hyperbilirubinemia in the current study and in A5257 were notably higher than in other cohort and clinical studies. It is unclear why this was the case for either study, although in the current study, the large proportion of patients of Hispanic origin (67.7%), for whom homozygosity for UGT1A1*28/*28 variant is overrepresented and known to increase rates of hyperbilirubinemia and discontinuation due to this cause,^24^ may have been influential. However, the rate of discontinuation due to hyperbilirubinemia in the current study was highest in German patients, for whom ocular icterus appears to have been a prominent reason. Given this inter-study and inter-country variation, further research is required to identify differences in baseline characteristics (such as ethnicity), genetics, laboratory parameters, and local clinical practices that are associated with an increased risk of patients discontinuing ATV/r due to hyperbilirubinemia.

Given that the current multivariate Cox proportional hazards analysis indicated that the overall risk of treatment discontinuation was lowest in Germany, factors other than treatment discontinuation due to hyperbilirubinemia-related reasons need to be considered. Of note, the proportion of patients at baseline with intravenous drug use was about threefold less in Germany than in Portugal or Spain (Table 1), which may have contributed to the higher rate of discontinuation in Portugal and Spain compared with Germany. Other possible reasons include regional variations in ARV treatment strategies. For example, it is common in Spain to simplify therapy in patients who achieve virologic suppression (as reflected by the highest reporting of the ‘Medical decision/Regimen simplification’ reason for discontinuation of ATV/r by Spanish investigators). Thus, higher rates of regimen simplification, rather than failure of the ATV/r regimen per se, may have contributed to the fact that the highest rates of discontinuation were observed in Spain (Table 3). In addition, a higher proportion of patients in Portugal and Spain than in Germany did not provide consent to continue into the one-year extension to the follow-up period (Table 3). Because the protocol amendment mandated that these patients should be considered as having discontinued ATV/r, this undoubtedly contributed to the higher rate of ATV/r discontinuation in Portugal and Spain. Had these patients provided consent, it is likely that some would have remained on treatment; therefore, the rate of ATV/r discontinuation in this study may have been overestimated.

In common with all observational studies, this study has acknowledged limitations. While the study sought to recruit patients from a wide variety of participating sites and consecutive clinic attendees were enrolled, it is not possible to state that the population studied here is representative of the general population of patients with HIV-1 infection commencing ARV therapy. Factors such as motivation to participate, reliability and completeness of historical and ongoing data collection, and ability to enroll sufficient numbers of patients within a set recruitment period were key issues contributing to the site selection process. It is possible, therefore, that patients in this study received a higher standard of clinical care leading to superior outcomes than that associated with non-participating centers. The observational and mixed retrospective–prospective study design meant that only historical data were collected for some patients, whereas for others the data collected were primarily prospective, and for both types of data collection, the length of follow-up varied. As a consequence, events may have been underreported during the retrospective phase. In common with all observational studies, bias from missing data or patients having been lost to follow-up cannot be excluded. In contrast to randomized trials where all AEs are systematically reported and assessed for causality, AEs could have been underreported. In addition, establishing whether there were causal relationships with ATV/r regimens was difficult owing to the lack of a comparator regimen in the current study.

Despite these methodological limitations, this study followed a large number of patients from different European countries over an extended period beyond that normally associated with randomized trials. In addition, the lack of narrow selection criteria, as commonly occurs in randomized trials, enabled the long-term outcomes of ATV/r-based regimens in a real-life clinical setting to be evaluated in patients who may have been ineligible for participation in randomized controlled trials.

To conclude, in a real-life clinical setting over five years, ATV/r-based regimens in treatment-naïve patients with HIV-1 infection showed sustained virologic suppression, an absence of treatment-emergent major PI resistance mutations at virologic failure, a lower overall rate of treatment discontinuation compared with other similar cohort studies examining alternative regimens, a long-term safety profile consistent with that previously observed in clinical trials, and no evidence of a significant decline in renal function for up to five years. ATV/r is an effective and well-tolerated therapeutic option for treatment-naïve patients with HIV-1 infection.

## Conflicts of interest

Eugénio Teófilo has received honoraria for attendance at advisory boards and investigator grants for clinical trials from Abbvie, Bristol-Myers Squibb, Gilead Sciences, Janssen-Cilag, Merck Sharp & Dohme, and ViiV Healthcare. Nuno Rocha-Pereira has received honoraria to attend meetings and congresses both in general infectious diseases medicine and in HIV medicine from Abbvie, Bristol-Myers Squibb, Gilead Sciences, Janssen-Cilag, Merck Sharp & Dohme, and ViiV Healthcare. Birger Kuhlmann declares no conflicts of interests. Antonio Antela has received investigator grants for clinical trials, and honoraria for attendance at advisory boards or for speaker engagements from Abbvie, Bristol-Myers Squibb, Gilead Sciences, GlaxoSmithKline, Janssen-Cilag, Merck Sharp & Dohme, and ViiV Healthcare. Heribert Knechten has received investigator grants from Abbott and Octapharma, and has received investigator grants and personal fees from AbbVie, Biotest, Bristol-Myers Squibb, Boehringer Ingelheim, Gilead Sciences, Janssen-Cilag, Merck Sharp & Dohme, and ViiV Healthcare. Jesús Santos has carried out consultancy work for Bristol-Myers Squibb, Gilead Sciences, and Janssen-Cilag; he has received monetary payments for giving talks from Bristol-Myers Squibb, Gilead Sciences, Janssen-Cilag, and Merck Sharp & Dohme, and has received payments for the development of educational presentations for Bristol-Myers Squibb, Gilead Sciences, Janssen-Cilag, and Merck Sharp & Dohme. Maria Jesús Jiménez-Expósito is an employee of Bristol-Myers Squibb.

## Adherence to ethics and reporting requirements

This study was conducted in accordance with the International Society for Pharmacoepidemiology (ISPE) Guidelines for Good Epidemiology Practices, the ethical principles originating from the Declaration of Helsinki, and local regulatory requirements.

## Study registration

Registered at clinicaltrials.gov (NCT01236235).
